# Different Secretory Activity of Articular and Subcutaneous Adipose Tissues from Rheumatoid Arthritis and Osteoarthritis Patients

**DOI:** 10.1007/s10753-018-0901-9

**Published:** 2018-10-02

**Authors:** Magdalena Plebańczyk, Anna Radzikowska, Tomasz Burakowski, Iwona Janicka, Urszula Musiałowicz, Anna Kornatka, Włodzimierz Maśliński, Ewa Kontny

**Affiliations:** Department of Pathophysiology and Immunology, National Institute of Geriatrics, Rheumatology and Rehabilitation, Spartanska 1, 02-637 Warsaw, Poland

**Keywords:** rheumatoid arthritis, osteoarthritis, articular adipose tissue, subcutaneous adipose tissue, cytokines, chemokines

## Abstract

Rheumatoid arthritis (RA) and osteoarthritis (OA) are characterized by joint and systemic high- or low-grade inflammation, respectively. Adipose tissue (AT) may contribute to the pathogenesis of these diseases. To address this issue, we investigated whether basal and pro-inflammatory cytokine (IL-1β)-triggered release of adipocytokines (TNF, IL-6, IL-10, IL-1Ra, TGFβ, CCL2/MCP-1, CCL5/RANTES, MMP-3) from subcutaneous (ScAT) and intraarticular (AAT) adipose tissues of RA and OA patients mirror differences between these diseases in an intensity of systemic and local inflammation. We found that in both diseases basal adipocytokine release was usually higher from AAT than ScAT, reflecting stronger local than systemic inflammation. However, ScAT secreted considerable amounts of pro- and anti-inflammatory factors as well. Spontaneous secretion of some adipocytokines (MMP-3 and/or TNF, CCL2/MCP-1, IL-1Ra) was higher in osteoarthritis than rheumatoid ATs and probably caused by weaker anti-inflammatory treatment of OA patients. By contrast, reactivity of ATs to IL-1β was significantly lower in OA than RA and IL-1β antagonist (IL-1Ra) could be responsible for this because we found its overproduction in OA ATs. Interestingly, higher reactivity of ScAT than AAT to IL-1β was a characteristic for OA while reactivity of rheumatoid ScAT and AAT to this stimulus was equal. We conclude that differences between OA and RA in reactivity of AAT and ScAT to pro-inflammatory stimulus mimicking *in vivo* condition reflect dissimilarity in an intensity of disease-specific inflammation and thus support contribution of ATs to these pathological processes. Moreover, we propose that more efficient anti-inflammatory mechanism(s) are preserved in ATs of OA than RA patients.

## INTRODUCTION

Rheumatoid arthritis (RA) and osteoarthritis (OA) are common joint diseases affecting millions of people worldwide [1, 2]. Although both diseases are characterized by joint and systemic inflammation, their pathogenesis is different. Rheumatoid arthritis is a destructive autoimmune disorder triggered by combination of genetic and environmental factors, characterized by chronic inflammation, supported by cells and factors of innate and adaptive immunity, resulting in a clinical picture—synovitis, cartilage, and bone damage as well as high-grade systemic inflammation which affects many organs outside the joint and causes complications such as cardiovascular disease [1, 3, 4]. Osteoarthritis is a frequently occurring musculoskeletal disorder causing stiffness, swelling, instability, and pain of the joints [5]. Risk factors are numerous, including age, female gender, genetic predisposition, anatomical structure, joint injuries, obesity, or metabolic syndrome [2, 5, 6]. Osteoarthritis often develops due to mechanical overloading of the joints accompanied by an imbalance between tissue damage and repair [7]. It is characterized by cartilage degradation, subchondral bone sclerosis, osteophyte formation as well as some degree of synovitis that may be either primary or secondary symptom [8, 9]. The disease affects also the soft tissue structures (joint capsule, menisci, ligaments) and is characterized by low-grade systemic inflammation [2, 7, 10]. Despite the progress in the treatment, both diseases are still incurable and lead to joint destruction and disability [2, 11–14].

Accumulating data suggest contribution of the white adipose tissue (WAT) to the pathogenesis and/or progression of different disease, including RA and OA [6, 15]. Adipose tissue, recognized now as the main body endocrine organ, contains various cell types (adipocytes, fibroblasts, macrophages, and lymphocytes) as well as components of the vascular and nervous systems [6, 15, 16]. These cells produce many biologically active substances (adipocytokines), including pro- and anti-inflammatory cytokines, classical adipokines, growth factors, complement components, and many others that act in a para-, endo-, and autocrine ways and control the metabolism and immune processes in antagonistic or synergistic manner [17–19]. Fat depots are heterogeneous in respect of body locations, features and functional characteristics, and their secretory activity is modified by inflammatory environment [20]. There are data suggesting contribution of intraarticular fat pad (IPFP, or Hoffa’s fat pad) and abdominal subcutaneous fat tissue to joint and systemic inflammation, respectively [6, 15, 21, 22].

The aim of present work was to further support contribution of WAT to rheumatic disease pathology by investigating whether basal and pro-inflammatory cytokine-triggered secretory activities of subcutaneous (ScAT) and intraarticular (AAT) adipose tissues obtained from OA and RA patients reflect differences between these diseases in an intensity of systemic and local inflammation and contribute to these pathological processes.

## MATERIALS AND METHODS

### Patients

Tissue specimens were obtained from OA [*n* = 44; female (F)/male (M) = 36/8; age = 62 (mean) (35–71) (min–max)] and RA [*n* = 43; F/M = 35/8; age = 54 (31–70)] patients at the time of total knee joint replacement surgery performed as a part of clinical care. Adipose tissue samples were taken from the Hoffa’s infrapatellar fat pad (AAT) and from the site of skin closure with sutures (ScAT). All patients signed an informed consent. The study was approved by the National Institute of Geriatrics, Rheumatology and Rehabilitation Ethics Committee. Patients’ characteristics are summarized in Table 1. OA patients were treated with non-steroidal anti-inflammatory drugs (NSAIDs) while RA patients were given disease modifying drugs (DMARDs) and/or glucocorticosteroids, none received biological therapy.

**Table 1:** Baseline Characteristics of Patients

	OA (*n* = 40)	RA (*n* = 39)	*p* value
Age (years)	63 (50–71)	56 (31–70)	0.00001
Female patients (*n*)	33	34	
Male patients (*n*)	7	5	
Disease duration (months)		192 (36–360)	
CRP (mg/l)	5 (0–16)	19 (5–85)	0.027
Weight (kg)	86 (62–143)	71 (44–100)	0.0003
Height (cm)	162 (150–177)	163 (156–180)	0.72
BMI	32.5 (24.1–45.1)	26.7 (17.6–37.7)	0.000005
	Underweight (0.0%)	Underweight (2.6%)	
	Normal (2.6%)	Normal (35.9%)	
	Overweight (41%)	Overweight (33.3%)	0.17
	Obese (56.4%)	Obese (28.2%)	0.001

Except where indicated otherwise values are the mean (minimum–maximum values); *p* < 0.05 statistically significant differences between OA and RA; *OA* osteoarthritis; *RA* rheumatoid arthritis; *CRP* C-reactive protein; *BMI* body mass index; *BMI*: < 18,5 underweight; 18.5–24.99 norm; 25.0–29.99 overweight; > 30.0 obesity

### Tissue and Cell Culture

Tissue samples were separated and processed within 2 h after surgery. Both tissues were cut with scissors in a Petri dish into small (about 8–10 mg) pieces, filtered through sterile gauze and washed with 100 ml of phosphate-buffered saline (PBS, Lublin). Then, the tissues were gently shaken in 20 ml of PBS in a plastic flat bottles (Nunc, 75 cm^2^, filter cap) for 10 min, centrifuged for 1 min at 290×*g* at room temperature, filtered and washed again with 100 ml of PBS, and finally weighed. Tissue explants were pre-cultured for 26 h in 24-well plate (100 mg/ml of culture medium/well) at 37 °C and 5% CO_2_. During this time, culture medium (CM, Dulbecco’s modified eagle medium - DMEM from Gibco, supplemented with 100 mg/ml kanamycin) was changed three times (after 1, 18, and 26 h). Then tissues were cultured for 24 h in CM alone (negative control) or in the presence of recombinant human IL-1β (1 ng/ml; R&D Systems, Minneapolis, MN, USA). Concentrations of pro-inflammatory (interleukin (IL)-6, tumor necrosis factor – TNF) and anti-inflammatory (IL-10, interleukin 1 receptor antagonist – IL-1Ra, transforming growth factor β-TGFβ) cytokines, chemokines (CCL2/MCP-1, CCL5/RANTES) and metalloproteinase 3 (MMP-3) were measured in culture supernatants by specific ELISA.

### Enzyme-Linked Immunosorbent Assays

The ELISAs were done using commercially available ELISA sets: DuoSet from R&D Systems (Minneapolis, MN, USA) for IL-1β, IL-1Ra, TGFβ, CCL2/MCP-1, CCL5/RANTES, MMP-3; Ready-SET-Go from eBioscience (San Diego, CA, USA) for IL-10 and TNF, while ELISA for IL-6 was done as previously described [21].

### Statistical Analysis

Data were analyzed using Statistica vol. 10.0 software (Stat Soft Inc., Tulsa, OK, USA). The normality of data distribution was assessed by Shapiro-Wilk test. All data (except MMP-3 in OA AAT and CCL2/MCP-1 in RA AAT) were not normally distributed. The Mann-Whitney U test was used for comparisons between AAT and ScAT tissues and between OA and RA groups, while the Wilcoxon test was applied to assess the effect of IL-1β stimulation (paired samples from the same patients). Differences were considered as statistically significant for *P* values < 0,05. Correlation was assessed using a Spearman’s Rank two-tailed test and *R* value is shown.

## RESULTS

### Characteristic of the Patients

Comparison of the study groups (Table 1) shows that OA patients were older, had higher body weight and body mass index (BMI). They were either obese or overweight and only one OA patient had normal BMI. In contrast, the prevalence of normal BMI, overweight and obesity in RA group was similar (about 30% each) and there was one underweight patient. However, we failed to find any correlation between the basal secretory activity of tested adipose tissues and patients’ body composition. The only exception, observed in OA group, was weak inverse correlation of IL-6 produced by ScAT with BMI (*R* = −0,46; *p* = 0,0049). Despite the presence of significant differences in patients’ age in studied groups, there was also no correlation of cytokine and chemokine production with age (data not shown). However, RA patients were characterized by higher serum concentration of C-reactive protein (CRP) than OA patients.

### Basal Secretory Activity of Articular and Subcutaneous Adipose Tissues Obtained from RA and OA Patients

**Fig. 1.**
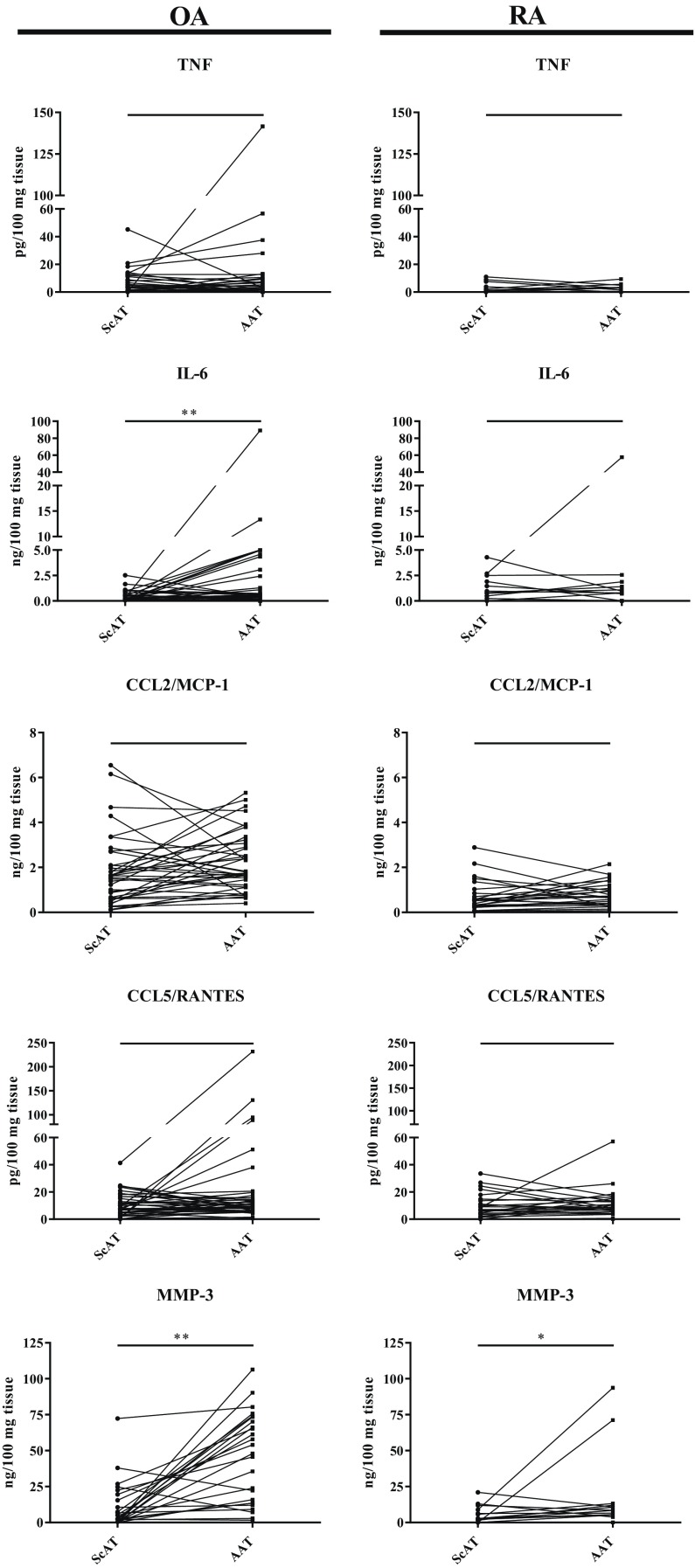
Basal secretion of proinflammatory cytokines and chemokines by articular (AAT) and subcutaneous (ScAT) adipose tissues obtained from osteoarthritis (OA) and rheumatoid arthritis (RA) patients. Tissues were cultured for 24 h in culture medium. Concentrations of proinflammatory cytokines (IL-6, TNF), chemokines (CCL2/MCP-1, CCL5/RANTES) and metalloproteinase (MMP-3) were measured in culture supernatants by specific ELISAs. Data are expressed as the level of adipo(cyto)kine production by 100 mg tissue and presented in pairs of tissues obtained from the same patient. Statistically significant differences between AAT and ScAT are shown (*p ≤ 0,05; **p ≤ 0,01). TNF tumor necrosis factor; IL interleukin; CCL2/MCP-1 C-C motif chemokine ligand 2/monocyte chemoattractant protein 1; CCL5/RANTES C-C motif chemokine ligand 5/regulated on activation, Normal T cell expressed and secreted; MMP-3 matrix metalloproteinase-3.

**Fig. 2:**
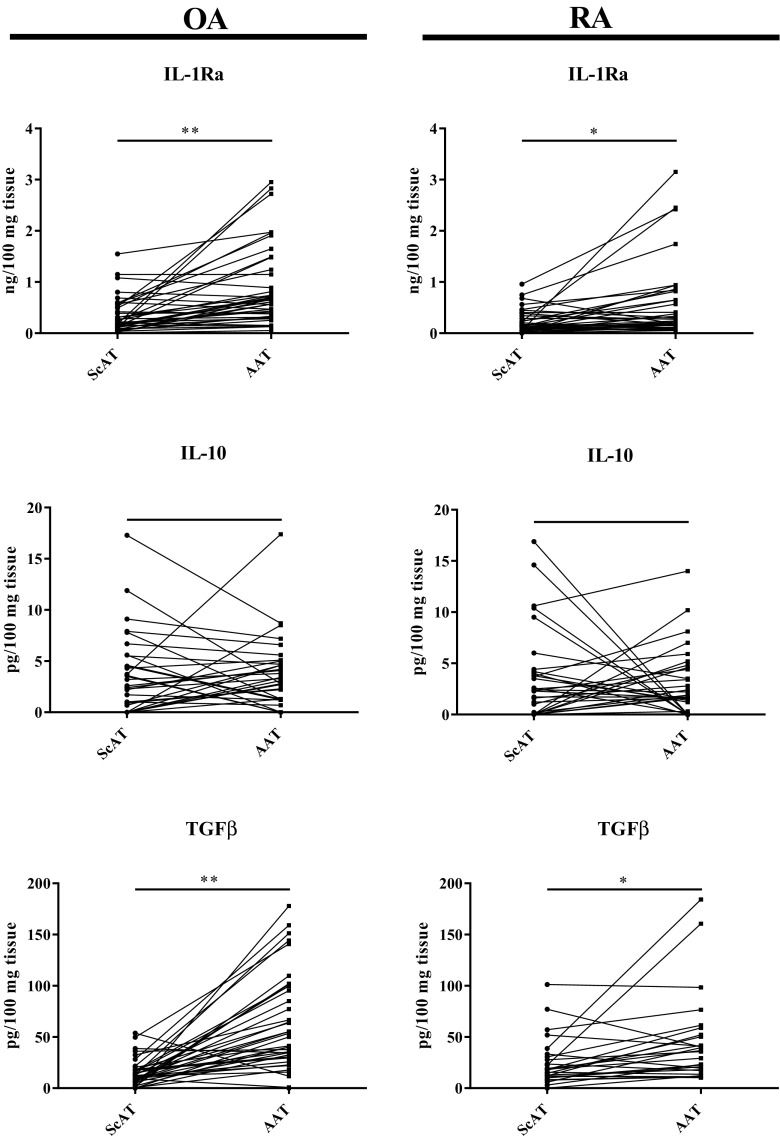
Basal secretion of anti-inflammatory cytokines by articular (AAT) and subcutaneous (ScAT) adipose tissues obtained from osteoarthritis (OA) and rheumatoid arthritis (RA) patients. Tissues were cultured for 24 h in culture medium. Concentrations of anti-inflammatory cytokines (IL-1Ra, IL-10, TGFβ) were measured in culture supernatants by specific ELISAs. Data are expressed as the level of cytokine production by 100 mg tissue and presented in pairs of tissues obtained from the same patient. Statistically significant differences between AAT and ScAT are shown (*p ≤ 0.05; **p ≤ 0.01). IL interleukin; IL-1Ra interleukin 1 receptor antagonist; TGFβ transforming growth factor beta.

In both study groups, basal secretory activity of AAT and ScAT was heterogeneous and characterized by the release of low (TNF, IL-10), moderate (TGFβ, CCL5/RANTES) or high (IL-6, CCL2, MMP-3, IL-1Ra) quantity of tested factors (Figs. 1 and 2). Comparison of spontaneous secretory activity of both adipose tissues showed some degree of inter-patient variability. However, irrespective of the type of rheumatic disease, AAT produced more connective tissue destructive enzyme – MMP-3 (Fig. 1) and anti-inflammatory cytokines (IL-1Ra and TGFβ) (Fig. 2). In addition, in OA, but not in RA patients, AAT secreted more IL-6 than corresponding ScAT explants (Fig. 1). Comparison of basal secretory activity of the same type of adipose tissue between the study patients’ groups revealed that OA ScAT produced more pro-inflammatory (TNF and CCL2/MCP-1) as well as anti-inflammatory (IL-1Ra) factors than RA ScAT, except TGFβ which was secreted in larger quantity by rheumatoid tissue (Table 2). Similar differences were observed between patients’ group in basal secretory activity of AAT (Table 3). However, in the latter case, adipose tissue of OA patients secreted also more MMP-3 and there was no significant difference between patients’ group in TGFβ secretion.

**Table 2:** Comparison of Spontaneous Adipocytokine Secretion by Subcutaneous Adipose Tissue Explants Obtained from Osteoarthritis (OA) and Rheumatoid Arthritis (RA) Patients

Adipocytokines	OA	RA	p value
TNF (pg/100 mg tissue)	1.3 (0.0–45.2)	0.0 (0.0–11.0)	0.0014
IL-6 (ng/100 mg tissue)	0.2 (0.0–2.52)	0.76 (0.0–4.29)	0.2
IL-1Ra (ng/100 mg tissue)	0.2 (0.01–1.55)	0.13 (0.0–0.96)	0.049
IL-10 (pg/100 mg tissue)	2.3 (0.0–17.3)	1.2 (0.0–16.9)	0.34
TGFβ (pg/100 mg tissue)	10.5 (0.0–53.7)	17.9 (0.0–101.3)	0.019
CCL2/MCP-1 (ng/100 mg tissue)	1.63 (0.11–6.55)	0.53 (0.03–2.89)	0.00023
CCL5/RANTES (pg/100 mg tissue)	7.8 (0.0–41.4)	7.1 (0.0–33.6)	0.76
MMP-3 (ng/100 mg tissue)	3.02 (0.0–72.39)	2.78 (0.0–29.97)	0.66

Values are the median (min–max); *p* < 0.05 statistically significant differences between OA and RA; *TNF* tumor necrosis factor; *IL* interleukin; *IL-1Ra* interleukin 1 receptor antagonist; *TGFβ* transforming growth factor beta; *CCL2/MCP-1* C-C motif chemokine ligand 2/monocyte chemoattractant protein 1; *CCL5/RANTES* C-C motif chemokine ligand 5/regulated on activation, normal T cell expressed and secreted; *MMP-3* matrix metalloproteinase-3

**Table 3:** Comparison of Spontaneous Adipocytokine Secretion by Articular Adipose Tissue Explants Obtained from Osteoarthritis (OA) and Rheumatoid Arthritis (RA) Patients

Adipocytokines	OA	RA	p value
TNF (pg/100 mg tissue)	1.9 (0.0–141.6)	0.0 (0.0–9.4)	0.0015
IL-6 (ng/100 mg tissue)	0.6 (0.0–89.14	0.78 (0.0–57.6)	0.46
IL-1Ra (ng/100 mg tissue)	0.65 (0.05–2.95)	0.22 (0.05–3.15)	0.0019
IL-10 (pg/100 mg tissue)	2.8 (0.0–17.4)	1.6 (0.0–14.0)	0.098
TGFβ (pg/100 mg tissue)	45.7 (0.0–177.9)	35.8 (10.34–184.3)	0.11
CCL2/MCP-1 (ng/100 mg tissue)	1.8 (0.4–5.32)	0.67 (0.08–2.14)	0.0000003
CCL5/RANTES (pg/100 mg tissue)	10.0 (0.6–231.7)	8.4 (0.0–57.1)	0.23
MMP-3 (ng/100 mg tissue)	47.98 (1.65–106.36)	8.39 (0.0–93.68)	0.02

Values are the median (min-max). Explanations as in Table 2

### Impact of Pro-inflammatory Stimulus on Adipose Tissue Activity

**Fig. 3.**
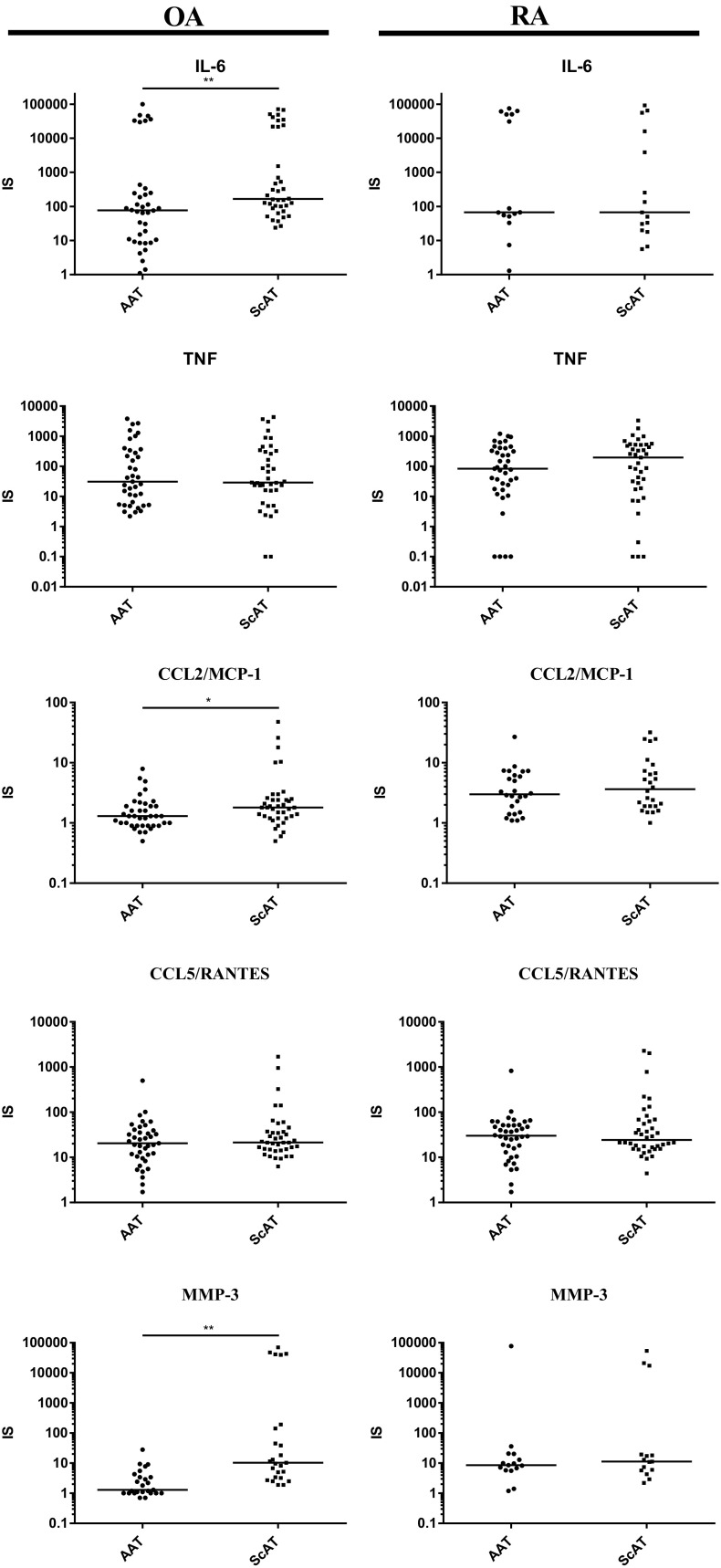
Reactivity of articular (AAT) and subcutaneous (ScAT) adipose tissues from osteoarthritis (OA) and rheumatoid arthritis (RA) patients to proinflammatory stimulus- secretion of proinflammatory cytokines (IL-6, TNF), chemokines (CCL2/MCP-1, CCL5/RANTES) and metalloproteinase MMP-3. Tissues were cultured for 24 h in culture medium alone (control) or in the presence of human recombinant IL-1β (1 ng/ml). Concentrations of tested adipocytokines were measured in culture supernatants by specific ELISAs. Effect of IL-1β stimulation was analyzed as stimulation to control ratio (IS, index of stimulation). Statistically significant differences between AAT and ScAT are shown (*p ≤ 0.05; **p ≤ 0.01); other explanations as in Fig. 1.

**Fig. 4:**
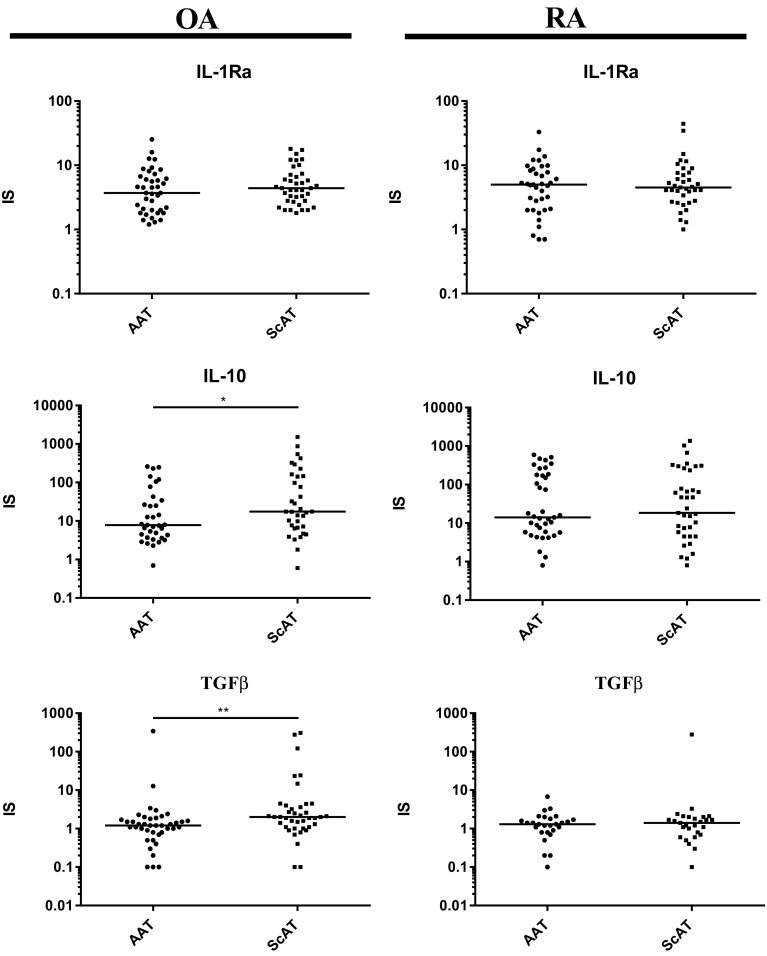
Reactivity of articular (AAT) and subcutaneous (ScAT) adipose tissues from osteoarthritis (OA) and rheumatoid arthritis (RA) patients to proinflammatory stimulus- secretion of anti-inflammatory cytokines (IL-1Ra, IL-10, TGFβ). Tissues were cultured for 24 h in culture medium alone (control) or in the presence of human recombinant IL-1β (1 ng/ml). Concentrations of tested cytokines were measured in culture supernatants by specific ELISAs. Effect of IL-1β stimulation was analyzed as stimulation to control ratio (IS, index of stimulation). Statistically significant differences between AAT and ScAT are shown (*p ≤ 0.05; **p ≤ 0.01); other explanations as in Fig. 2.

Reactivity of adipose tissues to pro-inflammatory stimulus was assessed as the ratio of adipocytokine secretion in IL-1β-treated to untreated cultures (index of stimulation, IS). As predicted, the secretion of all analyzed factors (except TGFβ released by ScAT obtained from RA patients) raised markedly upon IL-1β stimulation (Table 4). In OA patients, ScAT turned out to be much more reactive to a pro-inflammatory stimulus than AAT. In detail, higher increase of some pro-inflammatory (IL-6, CCL2/MCP-1, MMP-3) (Fig. 3) as well as anti-inflammatory (IL-10, TGFβ) (Fig. 4) factors’ secretion by ScAT than AAT was observed while up-regulation of other cytokines release was similar in both OA tissues. By contrast, in AAT and ScAT from RA patients, IL-1β-triggered increase of all tested factors secretion was similar (Figs. 3 and 4).

**Table 4:** Comparison of Reactivity of Articular Adipose Tissues (AAT) and Subcutaneous Adipose Tissues (ScAT) from Osteoarthritis (OA) and Rheumatoid Arthritis (RA) Patients to Proinflammatory Stimulus (IL-1β)

Adipocytokines	AAT			ScAT		
	OA	RA	*p* value	OA	RA	*p* value
TNF	31.2 (2.2–3838. 0)	84.1 (0.0–1210.0)	0.41	29.1 (0.0–4356.7)	197.0 (0.0–3330.0)	0.16
IL-6	76.6 (1.1–99,503.2)	67.0 (1.3–74,758.8)	0.32	165.0 (23.7–71,380	67.4 (5.6–91,924.5)	0.20
IL-1Ra	3.7 (1.2–25.3)	5.0 (0.7–33.0)	0.43	4.4 (1.8–18.0)	4.6 (1.0–44.3)	0.55
IL-10	7.8 (0.7–260.1)	14.0 (0.0–592.0)	0.09	17.6 (0.6–873.5)	18.4 (0.0–1360.0)	0.79
TGFβ	1.2 (0.0–343.0)	1.3 (0.1–6.8)	0.73	2.0 (0.0–308.3)	1.4 (0.0–279.6)	0.02
CCL2/MCP-1	1.3 (0.9–7.9)	3.0 (1.1–26.8)	0.00003	1.8 (0.5–47.7)	3.7 (1.0–31.9)	0.003
CCL5/RANTES	21.6 (1.7–499.3)	36.9 (2.5–823.6)	0.08	21.8 (6.3–3361.0)	42.1 (4.4–2287.6)	0.24
MMP-3	1.3 (0.7–28.0)	8.6 (1.2–77,140.3)	0.0002	10.3 (1.9–70,527.4)	11.3 (2.2–54,214.2)	0.91

Data are shown as index of stimulation (IS). Values are the median (min–max). Explanations as in Table 2

Moreover, upon IL-1β treatment, the up-regulation of CCL2/MCP-1 secretion in both rheumatoid tissues was significantly higher than in OA tissues. The same effect was observed in the case of MMP-3 production by AAT, but not ScAT. By contrast, we observed also higher increase of TGFβ release by ScAT obtained from OA than from RA patients (Table 4).

## DISCUSSION

Rheumatoid arthritis and osteoarthritis are characterized by local joint inflammation and systemic inflammation of high- and low-grade, respectively. Thus, disease-specific pro-inflammatory environment can affect both articular a well as subcutaneous adipose tissue located near the joint. Previous publications have indicated that WAT is a very active tissue-secreting adipocytokines that can participate in the development and course of inflammation not only inside the joint but also throughout the body in both RA and OA. These reports have shown that in most cases adipocytokine production is significantly higher in articular than in subcutaneous adipose tissues [21–24]. In order to broaden and systematize knowledge about contribution of adipose tissue to these diseases pathology, we decided not only to compare the secretory activity of both AAT and ScAT and to assess their ability to react to the inflammatory stimulus, but also to compare tissues obtained from patients with RA and OA. We assumed that we would find a number of differences between RA and OA in the activity of AAT and ScAT, reflecting dissimilarities of these diseases background, course and intensity of inflammation. Such information would be also useful in pointing out adipose tissue as a possible therapeutic target in these rheumatic diseases.

Consistently with previous reports [21, 22, 24], we found, both in RA and OA, higher spontaneous production of some factors (MMP-3, IL-1Ra, TGFβ) in AAT than ScAT (Figs. 1 and 2). MMP-3 is an enzyme involved in homeostatic wound healing as well as in pathological processes inside affected joints—its role in the degradation of joint cartilage is well known [22, 25]. The higher level of MMP-3 in AAT in both studied diseases is consistent with local destructive role of this enzyme. Higher production of anti-inflammatory TGFβ and IL-1Ra (natural IL-1β antagonist) may be an attempt to self-regulate and reduce local inflammation. In addition, we noticed higher level of IL-6 secretion by AAT than by ScAT in OA, but not RA, patients (Fig. 1). Other authors reported up-regulation of IL-6 in AAT in both diseases [6, 22, 26]. Differences in RA clinical activity and treatment of patients may explain this discrepancy. Interleukin 6 exerts a variety of effects on both innate and adaptive immune cells, is involved in the regulation of inflammation and lipid metabolism. It is estimated that about 35% of circulating IL-6 is derived from adipose tissue [27]. Elevated levels of this cytokine, correlated with joint destruction, were detected in the serum, synovial fluid and fibroblast-like synoviocytes (FLS) of RA patients [1, 3]. Also in OA up-regulated serum concentration of IL-6 was reported to be associated with the progression of inflammation [6, 28]. Our results showing in OA patients larger amounts of this pro-inflammatory cytokine in AAT than ScAT seem to reflect the presence of much stronger inflammation inside the joint than outside it. In RA, however, similar amounts of IL-6 in both tissues seem to mirror high-grade inflammation not only in the joint, but also in neighboring tissues, and suggest equal contribution of AAT and ScAT to support it (Fig. 1, Table 2 and 3). Despite above differences in basal secretory activities of tested adipose tissues, present results revealed ScAT located nearby inflamed joints as an active tissue, producing significant amounts of adipocytokines (Table 2). Therefore, we believe that it can contribute to the development, maintenance, and regulation of inflammation in both RA and OA.

We also found that both adipose tissues derived from OA patients produced spontaneously larger amounts of TNF and CCL2/MCP-1 than analogous rheumatoid tissues (Tables 2 and 3). The role of TNF in inflammatory processes is well understood—it is critical for the development of inflammatory response and triggers production of other pro-inflammatory cytokines, including IL-1β and IL-6. Like IL-1β, TNF enhances the synthesis of proteolytic enzymes, downregulates their inhibitors, and reduces production of articular tissue matrix macromolecules [23, 29]. In adipose tissue, TNF is synthesized by adipocytes and infiltrating macrophages [27]. TNF is obviously a critical factor involved in the pathogenesis of RA [1]. Its important role has also been confirmed in OA [29]. CCL2/MCP-1 in turn is a chemokine which among other activities attracts macrophages and T cells into adipose tissue [22]. In addition, we stated that AAT from OA patients secreted significantly more MMP-3 than respective rheumatoid tissue (Table 3). The above differences between OA and RA patients in basal secretory activities of AAT and ScAT may be caused by inequalities in the treatment, as RA patients received DMARDs and/or glucocorticosteroids while OA patients were given NSAIDs only. It is possible that weaker anti-inflammatory therapy in OA was not sufficient enough to control activity of tested tissues *in vivo*. Higher levels of some adipocytokines (TNF, CCL2/MCP-1) in ScAT from OA than RA patients can also result from different body compositions, as obesity was more frequent in OA than RA group (Table 1). Obesity is known to be associated with low-grade inflammation of adipose tissue [21]. However, we failed to find significant correlation between secretory activity of tested adipose tissues and BMI of the patients. Therefore, dissimilarity of the patients’ treatment is a more possible explanation.

Interestingly, we noticed an important difference in the anti-inflammatory cytokines secretion between diseases as well. Both adipose tissues from OA patients secreted more IL-1Ra compared to RA group (Tables 2 and 3). This antagonist is a potent inhibitor of action of IL-1β—cytokine endowed with strong ability to activate adipose tissue [29]. Rheumatoid ScAT secreted in turn greater quantity of TGFβ (Table 2). By contrast to IL-1Ra, TGFβ is a pleiotropic regulator of immune response, which controls differentiation and function of innate and adaptive immune cells. It plays an important role in the development of several T cell lineages, including regulatory T cells while suppresses Th1 and Th2 cells [30]. Thus, it is likely that in OA IL-1Ra overproduced by AAT and ScAT exerts overall protective anti-inflammatory effect on adipose tissue while in RA considerable amounts of ScAT-originating TGFβ restrict rather high-grade systemic inflammation.

Finally, to mimic *in vivo* milieu we evaluated and compared reactivity of AAT and ScAT from both patients’ groups to pro-inflammatory IL-1β. It is known that IL-1β is produced in inflamed joints by synovial cells and triggers production of TNF and other adipocytokines [22]. As expected, in our *in vitro* experiments, IL-1β caused significant increase of almost all analyzed factors in both AAT and ScAT of OA and RA patients (Table 4). Interestingly, in OA reactivity of ScAT to pro-inflammatory IL-1β was significantly higher than reactivity of AAT as a greater increase of pro-inflammatory IL-6, MMP- 3, and CCL2/MCP-1 as well as anti-inflammatory IL-10 and TGFβ secretion was observed in this tissue (Figs. 3 and 4). By contrast, in RA the reactivity of AAT and ScAT to IL-1β did not differ (Figs. 3 and 4). These results suggest that in osteoarthritis ScAT, located in an inflammatory environment of lower intensity than AAT, maintains a greater ability to respond to an inflammatory agent. In RA, we failed to find such differences probably because both AAT and ScAT are exposed to high-grade, chronic inflammation. Importantly, comparison of ScAT and AAT from OA *versus* RA patients revealed that rheumatoid tissues are characterized by higher reactivity to pro-inflammatory IL-1β, at least in the case of CCL2/MCP-1 and MMP-3 secretion (Table 4), giving support to critical role of this stimulus in RA pathology. As mentioned above, for both ScAT and AAT of OA patients high-basal secretion of IL-1Ra is a characteristic (Tables 2 and 3). Therefore, this unique disease-specific and inflammatory-protective feature may explain lower reactivity of osteoarthritis adipose tissues to IL-1β and consequently lower-grade inflammation, comparing with RA.

It should be underlined that our study has several limitations. Firstly, due to ethical reasons, we were unable to compare activity of articular and subcutaneous adipose tissues obtained from RA and OA patients to respective tissues from healthy donors. Thus, we could only characterize tissues from diseases with different intensity of systemic and local inflammation. Secondly, we focused only on the secretion of selected cytokines known to be important in analyzed diseases development and activity. As was mentioned in the introduction, white adipose tissue secretes many active factors. According to available knowledge classical adipokines are also considered as the key players in inflammation, also in rheumatic diseases [31]. Leptin, adiponectin, resistin, visfatin, and others have been reported to be involved in RA and OA pathogenesis by their impact on cartilage [32], synovium [33], bone, adipose mesenchymal stem cells [34], and various immune cells [31, 35]. Thus, our work is only a step for understanding the complex process in the patients’ body.

In conclusion, based on comparison between OA and RA patients in respect of ScAT and AAT secretory activity, we report that adipose tissues of OA patients are characterized by higher basal secretion of some pro- and anti-inflammatory adipocytokines but lower reactivity to inflammatory hit. We suppose that these differences may be caused by weaker anti-inflammatory treatment of OA patients and better control of osteoarthritis adipose tissues by endogenously produced IL-1Ra, respectively. In addition, we demonstrate that in both diseases ScAT located nearby affected joint is a rich source of pro- and anti-inflammatory factors. However, in OA, this tissue retains higher reactivity to pro-inflammatory stimulus than AAT while in RA reactivity of both adipose tissues is similar. We assumed that these differences reflect *in vivo* exposure of adipose tissues to inflammatory milieu of various grades.
